# Thyrotroph embryonic factor is downregulated in bladder cancer and suppresses proliferation and tumorigenesis via the AKT/FOXOs signalling pathway

**DOI:** 10.1111/cpr.12560

**Published:** 2018-12-04

**Authors:** Jianan Yang, Bin Wang, Han Chen, Xuhong Chen, Jing Li, Yanfei Chen, Daozhang Yuan, Shunsheng Zheng

**Affiliations:** ^1^ Department of Urologic Oncosurgery Affiliated Cancer Hospital & Institute of Guangzhou Medical University Guangzhou China; ^2^ Key Laboratory of Protein Modification and Degradation, the Department of Pathophysiology, School of Basic Medical Sciences Guangzhou Medical University Guangzhou China

**Keywords:** bladder cancer, FOXO, G1/S, proliferation, TEF

## Abstract

**Objectives:**

Thyrotroph embryonic factor (TEF) plays an important role in several different processes in normal human cells; however, its function in malignant cells has not been fully elucidated.

**Materials and methods:**

The mRNA levels of *TEF* in 408 bladder cancer (BC) samples from the Cancer Genome Atlas (TCGA) database were analysed in depth. Next, the expression of TEF in 7 BC cell lines was compared to that in normal bladder epithelial cells. The cell count, colony formation and anchorage‐independent growth assays as well as a nude mouse xenograft model were utilized to examine the effects of TEF on proliferation and tumorigenesis. Immunofluorescence staining, flow cytometry analysis and treatment with an AKT inhibitor were performed to explore the molecular regulation mechanisms of TEF in BC.

**Results:**

Analysis of TCGA data indicated that *TEF* mRNA was decreased in BC samples compared to that in normal bladder epithelial cells and correlated with the poor survival of BC patients. Additional experiments verified that the mRNA and protein expression of TEF were significantly decreased in BC cells compared to that in normal bladder epithelial cells. Upregulation of TEF expression significantly retarded BC cell growth by inhibiting the G1/S transition via regulating AKT/FOXOs signalling.

**Conclusion:**

Our results suggest that TEF might play an important role in suppressing BC cells proliferation and tumorigenesis.

## 1 INTRODUCTION

Bladder cancer (BC) is the second most common cancer of the genitourinary tract worldwide.^1^ The prognosis of patients with muscle‐invasive BC is poor given the currently available treatments.^2^ Conversely, most cases of non‐muscle‐invasive BC can be essentially cured when treated with a combination of surgical resection and chemotherapy at an early stage.^3^ Obviously, the key to improving BC patient prognosis is early diagnosis and treatment.^4^ However, traditional imaging techniques, such as ultrasonography, vesical radiography and magnetic resonance imaging, are of limited value for an early, definitive diagnosis of BC.^5^, ^6^ As the most reliable method currently available for the diagnosis of BC, cystoscopy can detect the size, number, shape, location and base and surrounding conditions of the tumour to confirm the pathological diagnosis.^8^, ^9^ Unfortunately, cystoscopy is an invasive diagnostic procedure, and its use is limited by the lack of suitable biomarkers for pathological staining. Currently, the identification of new markers is of substantial importance for enabling an early diagnosis of BC.^10^, ^11^ Although a number of molecules related to BC have been described in the literature, such as forkhead box M1 (FOXM1), collagen type V alpha 2 chain (COL5A2) and N‐myc, there are still no effective molecular targets for clinical diagnosis and therapy.^12^, ^13^ Thus, more specific and sensitive biomarkers and therapeutic targets need to be identified to improve the early diagnosis and prognosis of BC patients.

It is well known that the biological and molecular expression profiles of tumour cells are similar to those of embryonic cells but differ greatly from those of normal adult cells.^15^ Therefore, an increasing number of studies have been devoted to studying the molecular differences between normal adult and embryonic cells to identify targets, such as alpha fetoprotein (AFP), for the diagnosis and treatment of malignant cells.^16^ As a member of the proline and acidic amino acid‐rich (PAR) subfamily of basic region/leucine zipper (bZIP) transcription factors, which also includes albumin D box‐binding protein (DBP), human hepatic leucemia factor (HLF) and chicken vitellogenin gene‐binding protein (VBP), Thyrotroph embryonic factor (TEF) is expressed in a broad range of cells and tissues in adult animals.^17^, ^18^ TEF expression appears to be restricted to the developing anterior pituitary gland during embryonic development.^20^ In normal cells, TEF is an apoptotic regulator of hematopoietic progenitors and controls hematopoietic cell proliferation.^21^ In mouse fibroblasts, TEF has been reported to control actin distribution and cell shape.^22^ Furthermore, TEF has been reported to be involved in the development of various human diseases. For example, a study including 408 subjects using a linear regression model showed that TEF is associated with the Hamilton Rating Scale for Depression (HAMD) scores in patients with Parkinson's disease after adjusting for clinical variables.^23^ Using multivariate generalized linear models, the association between TEF and depressive symptoms further confirmed that TEF is closely related to the development of human disease.^24^ However, the role of TEF in malignant processes is still unknown.

In the present study, we demonstrate that *TEF* mRNA and protein expression are downregulated in BC cells and human BC tissues. Overexpressed TEF significantly inhibits the G1/S transition in the cell cycle as well as tumorigenesis via regulating the AKT/FOXOs signalling pathway. In addition, opposite results were observed in TEF‐silenced cells. These results suggest a potential role for TEF as a diagnostic marker and a valuable therapeutic target in BC.

## 2 MATERIALS AND METHODS

### 2.1 Microarray data processing and visualization

The RNA sequencing V2 profile dataset was downloaded on November 29th, 2014 from the Cancer Genome Atlas (TCGA) and contained 408 BC tissue and 19 adjacent normal bladder tissue samples. Profile data extractions were performed using Excel and MeV 4.9 (http://www.tm4.org/mev), and Gene Set Enrichment Analysis (GSEA) was performed using gsea 2.2.1 (http://www.broadinstitute.org/gsea).

### 2.2 Statistical analysis

spss version 13.0 (SPSS Inc, Chicago, IL) was used in all statistical analyses.^25^ The associations between the *TEF RNA* level and the clinicopathological characteristics of the TCGA data were analysed using the Chi‐squared test. Survival curves were plotted using the Kaplan‐Meier method and compared using the log‐rank test. A two‐tailed *P*‐value of <0.05 was considered statistically significant in all tests.

### 2.3 Cell lines and tissues

Primary normal bladder epithelial cells (Normal) and five paired BC tissues (ANT: adjacent normal tissues; T: tumor) were collected from the tumour resections of patients at the Department of Urologic Oncosurgery, Cancer Center of Guangzhou Medical University (PR China) in accordance with the rules and regulations concerning ethical research on human subjects in China. Samples were cultured in keratinocyte serum‐free medium (Invitrogen Life Technologies, Carlsbad, CA) supplemented with epithelial growth factor, bovine pituitary extract and antibiotics as previously described.^26^, ^27^ BC cell lines (SV‐HUC‐1, 5637, HT‐197, RT4, RT2, HT‐1376 and NBT‐2) were purchased from ATCC (Rockville, MD) and maintained in Dulbecco's modified Eagle’s medium (DMEM; Gibco, Rockville, MD) supplemented with 10% foetal bovine serum (HyClone, Logan, UT).

### 2.4 RNA extraction and real‐time quantitative PCR

Total RNA was extracted from BC cells, fresh tissues and the stably constructed cell lines using TRIzol reagent (Invitrogen Life Technologies) according to the manufacturer’s instructions. The RNA was then reverse transcribed and subjected to real‐time quantitative PCR (RT‐qPCR) as previously described.^26^ RT‐qPCR was performed using the Biosystems 7500 Sequence Detection system. The primers used were as follows: *TEF* (forward, 5′‐CTGCCTCACAACGACTCCTTTCTCT‐3′; reverse, 5′‐TCGCCTCTGTCTCCTCTTCACCATAG‐3′); *p21^Cip1 ^*(forward, 5′‐CGATGCCAACCTCCTCAACGA‐3′; reverse, 5′‐TCGCAGACCTCCAGCATCCA‐3′); *p27^Kip1^* (forward, 5′‐TGCAACCGACGATTCTTCTACTCAA‐3′; reverse, 5′‐CAAGCAGTGATGTATCTGATAAACAAGGA‐3′) and *GAPDH *(forward, 5′‐ACCACAGTCCATGCCATCAC‐3′; reverse, 5′‐TCCACCACCCTGTTGCTGTA‐3′). Expression data were normalized to the geometric mean of the housekeeping gene *GAPDH* to control for variability in the expression levels.

### 2.5 Western blot analysis

Western blot analysis was performed according to standard methods as previously described.^27^, ^28^ Total protein was extracted from cell pellets or fresh tissue after harvest. The blots were immunostained with primary and secondary antibodies. Anti‐TEF (1:500) was purchased from Abcam (Cambridge, MA); anti‐Ki67 (1:1000) was purchased from Cell Signalling (Danvers, MA); and other antibodies, including anti‐p‐AKT^Ser473^ (1:500), anti‐p‐AKT^Thr308^ (1:500), anti‐AKT (1:500), anti‐CDK4 (1:500), anti‐CDK6 (1:500,), anti‐cyclin D1 (1:500), anti‐p21^Cip1^ (1:200), anti‐p27^Kip1^ (1:200), anti‐p‐FOXO1^Ser256^ (1:500), anti‐FOXO1 (1:1000), anti‐p‐FOXO4^Ser197^ (1:500) and anti‐FOXO4 (1:500), were purchased from Sigma (St. Louis, MO). The membranes were stripped and reblotted with an anti‐α‐tubulin monoclonal antibody (1:1000; Abcam), which served as a loading control.

### 2.6 Vectors and retroviral infection

The *TEF* expression construct was generated by subcloning PCR‐amplified full‐length human *TEF* cDNA into the pMSCV‐retro‐puro vector (Clontech, Palo Alto, CA) using the forward primer 5′‐CCGCTCGAGATGAGCTGGCAGGTGGCCGAG‐3′ and reverse primer 5′‐CCGGAATTCTCACATTCTCATTTCAAAATATTTAATTTTGTCTG‐3′. The pSUPER.retro.puro plasmid (Oligoengine, Seattle, WA) was used as a clone carrier to endogenously downregulate *TEF* using two human shRNA sequences (RNAi1, CACCGGCCAGAGAAGAGAACAGATATTCAAGAGATATCTGTTCTCTTCTCTGGCC; RNAi2, AAAAGGCCAGAGAAGAGAACAGATATCTCTTGAATATCTGTTCTCTTCTCTGGCC), which were synthesized by Invitrogen. Retroviral production and infection were performed as described previously.^26^ The stable cell lines used in the present study were constructed from RT4 and HT‐1376 cells (RT4/HT‐1376‐TEF‐Vector [Vector1], RT4/HT‐1376‐TEF(TEF), RT4/HT‐1376‐TEF‐RNAi‐Vector [Vector2], RT4/HT‐1376‐TEF‐RNAi1 [RNAi1], RT4/HT‐1376‐TEF‐RNAi2 [RNAi2]). The reporter plasmid for quantitatively detecting the transcriptional activity of FOXOs was generated using the *pGL3*‐enhancer plasmid (Promega, Madison, WI) as described previously.^29^ According to the manufacturer’s instructions, perifosine (20 µmol/L; Abcam), a novel AKT inhibitor, was used to verify that AKT is involved in regulating the effects of TEF on proliferation. Water, the storage solution and diluent of perifosine, was used as experimental control. After 6 days of continuous treatment of perifosine, RT4‐TEF cells and HT‐1376‐TEF cells were used for other tests.

### 2.7 MTT assay

3‐(4,5‐Dimethyl‐2‐thiazolyl)‐2,5‐diphenyl‐2H‐tetrazolium bromide (MTT; Sigma) can be converted into blue‐purple crystalline formazan only by living cells, and the formazan is then deposited into the cells. The water‐insoluble formazan can be dissolved in dimethyl sulfoxide (DMSO), and the optical density of the formazan solution reflects the number of live cells. According to a previously described method, 2000 cells were seeded into six 96‐well plates in triplicate and allowed to attach and grow for 24 hour.^26^ At each time point, groups of cells were incubated with 100 μL of 0.5 mg/mL sterile MTT for 4 hour at 37°C. Then, the culture medium was removed, and 150 μL of DMSO (Sigma) was added. After shaking for 10 minute, the absorbance values were measured at 490 nm as the reference wavelength.

### 2.8 Flow cytometry analysis

Flow cytometry analysis was used to measure the DNA distribution and to identify the cell cycle of the tested cells. Cells were seeded at an initial density of 50 000 cells in 100‐mm dishes in the culture medium and allowed to attach for 24 hour. Then, the medium was replaced with a fresh medium containing 20 μmol/L perifosine or a vehicle, and the cells were cultured for 48 hour. Cells were harvested by trypsinization, washed with ice‐cold phosphate buffer solution (PBS, pH 7.4) and fixed in 80% ice‐cold ethanol in PBS. Cells were pelleted in a refrigerated centrifuge and resuspended in cold PBS. Then, the cells were incubated with bovine pancreatic RNase (working concentration 20 μg/mL; Sigma) at 37°C for 30 minute and stained with 20 μg/mL propidium iodide (Sigma). After incubation for 20 minute at room temperature, 20 000 cells were assayed using a FACSCanto II flow cytometer (BD Biosciences, San Jose, CA), and the data were analysed using the FLOWJO software (Tree Star, Inc, Ashland, OR). All experiments were performed in triplicate.

### 2.9 Colony formation assay

Colony formation assays reflect cell proliferation according to the number of clones formed by adherent cells. As previously described, 1000 cells/well were incubated in 6‐well plates. All stable cell lines were included in the colony formation assay.^26^ Ten days later, the colonies were fixed with 10% formaldehyde for 5 minute and then stained with 1.0% crystal violet for 30 seconds. All experiments were performed in triplicate for each cell line.

### 2.10 Anchorage‐independent growth assay

A soft agar, anchorage‐independent growth assay was used to determine the growth ability of malignant cells as a suspension. As previously described, 500 cells/well (RT4‐Vector1, RT4‐TEF, RT4‐vector2, RT4‐RNAi1, HT1376‐Vector1, HT1376‐TEF, HT1376‐vector2, HT‐1376‐RNAi1) were seeded in 2 mL of complete medium plus 0.3% agar (Sigma) for 12 days.^26^ All experiments were performed in triplicate for each cell line.

### 2.11 Bromodeoxyuridine labelling and immunofluorescence assay

Bromodeoxyuridine (BrdU) can be incorporated into the newly synthesized DNA of cells during the S phase and is commonly used to detect proliferating cells. All stable cell lines were routinely cultured at the initial density of 2000 cells per well in 24‐well plates with coverslips (Fisher, Pittsburgh, PA) placed inside the wells, and the cells were allowed to attach for 72 hour. The medium was replaced with a fresh medium containing 10 µmol/L BrdU, and the cells were cultured for 1 hour. The medium was removed and labelled, and the cells were washed twice in PBS. Then, the cells were fixed in 100% methanol (chilled at −20°C) for 5 minute, permeabilized with 0.2% TritonX‐100 for 10 minute, and then immunostained with an anti‐BrdU antibody (Upstate, Temecula, CA). A laser scanning microscope (Axioskop 2 plus; Carl Zeiss Co. Ltd., Jena, Germany) was used for imaging.

### 2.12 Xenograft model of BC in nude mice and immunohistochemical staining

Use of a xenograft model to better simulate the cell growth process in vivo and reflect the malignancy of tumours. The in vivo experiments were performed as previously described.^26^ All institutional and national guidelines for the care and use of laboratory animals were followed. Non‐obese diabetic/severe combined immunodeficiency (NOD/SCID) mice (4‐5 weeks old, 18‐20 g) were purchased from the Guangdong Medical Laboratory Animal Center (Guangzhou, Guangdong, China). Cells (5 000 000) were injected into the left (HT‐1376‐pMSCV‐vector) and right abdomen (HT‐1376‐TEF) of each mouse. The tumours were examined every 3 days for 18 days. On Day 18, the animals were euthanized, and the tumours were excised and weighed. Each excised tumour was cut in half, with one half being used for Western blot, and the other half being embedded in paraffin, sliced and subjected to immunohistochemical (IHC) staining. According to the manufacturer’s instructions, tumour samples were stained with TEF (1:200) and Ki67 (1:800) antibodies using an anti‐rabbit HRP/DAB detection kit (Abcam).

## 3 RESULTS

### 3.1 TEF is downregulated in BC and correlates with poor patient survival

**Figure 1:**
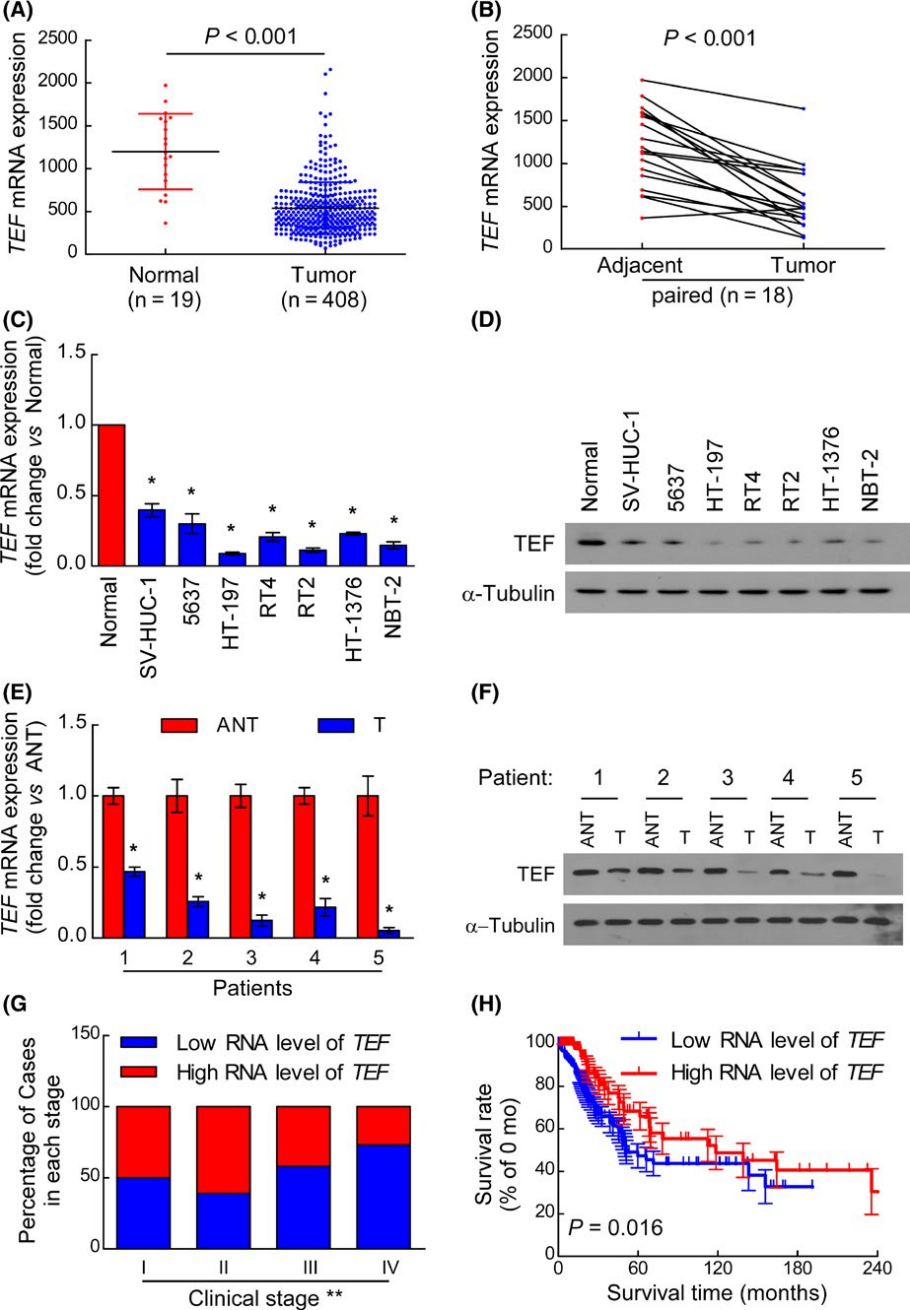
Thyrotroph embryonic factor (TEF) expression is downregulated in BC. (A) The mRNA level of *TEF* was frequently downregulated in 408 BC tissues (Tumour) compared to that in 19 normal bladder epithelial samples (Normal) in the TCGA database. Values are expressed as the mean ± SD *P *< 0.001 for Tumour vs Normal. (B) The RNA levels of TEF were markedly decreased in 18 paired BC tissues (Tumour) compared to those in adjacent normal tissues (Adjacent) from 18 patients in the TCGA database. Values are expressed as the mean ± SD *P *< 0.001 for Tumour vs Adjacent. (C) RT‐qPCR analysis of TEF expression in normal epithelial cells (Normal) and BC cell lines, including SV‐HUC‐1, 5637, HT‐197, RT4, RT2, HT‐1376 and NBT‐2. Three independent experiments were conducted. (D) Western blot analysis of TEF expression in Normal and BC cell lines. (E) The *TEF *mRNA expression level was downregulated in five paired BC tissues (T) compared to that in the corresponding adjacent normal tissue (ANT). The average *TEF* mRNA level was normalized to the expression of GAPDH. *P < 0.05 for T vs ANT. (F) The expression of TEF was downregulated in five paired BC tissues (T) compared to that in the corresponding adjacent normal tissue (ANT). Three independent experiments were conducted. (G) After Chi‐Square testing all 400 patient samples from the TCGA database according to their clinical stage, in which samples were categorized as having either high or low *TEF* expression, the RNA level of *TEF* was found to negatively correlate with the BC clinical stage. **P < 0.01 for Stage I vs Stage II vs Stage III vs Stage IV. (H) Kaplan‐Meier overall survival curves for all 294 patients with available survival time data are depicted. *P* = 0.016 for low levels of TEF RNA vs high levels of TEF RNA

Analysing 408 BC cases from TCGA database showed that the mRNA expression of *TEF *was downregulated in BC samples compared to that in adjacent normal tissue samples (19 cases; *P *< 0.001, Figure 1A). To account for individual differences, the mRNA expression of *TEF* in a total of 18 paired tissues further demonstrated that the level of TEF mRNA expression was significantly downregulated in all 18 bladder tumour tissues compared to that in the matched adjacent normal tissues (ANT, *P* < 0.001; Figure 1B). To verify this finding, seven cultured BC cell lines (SV‐HUC‐1, 5637, HT‐197, RT4, RT2, HT‐1376 and NBT‐2) and five pairs of clinical bladder specimens were further tested at both the mRNA and protein level, and the results were consistent with the previous findings using both types of samples. RT‐qPCR analysis verified that the mRNA level of *TEF* was indeed downregulated in cultured BC cell lines compared to that in normal epithelial cells (Normal, Figure 1C). Meanwhile, the mRNA level of *TEF* was also downregulated in five fresh BC tissues (T) compared to that in the paired adjacent normal tissue (ANT; Figure 1E). Western blot analysis revealed that TEF protein expression was also downregulated in malignant BC cells (or tissues) compared to that in non‐malignant cells (or tissues, Figure 1D,F). Taken together, these results strongly indicate that TEF is downregulated in human BC.

To investigate the significance of *TEF *downregulation in BC, data from the TCGA were categorized and analysed in depth. Using the mean value as a cut‐off, all 400 patients with clinical staging data were categorized into one of two groups based on the *TEF* RNA level in their tumours: a high *TEF* RNA group and a low *TE*F RNA group. As shown in Figure 1G, the RNA expression of *TEF* was higher in early‐stage tumours (stages I‐II) and was lower in advanced‐stage tumours (stages III‐IV, *P* < 0.01). Kaplan‐Meier survival curves were plotted for the 294 patients with available survival data, and the results showed that the overall survival of patients with low *TEF* RNA level was significantly shorter than that of patients with high *TEF* RNA level (Figure 1H, *P *= 0.016). Collectively, these results indicate that downregulation of TEF in BC patients correlates with poor survival.

### 3.2 TEF regulates the proliferation of BC cells

**Figure 2:**
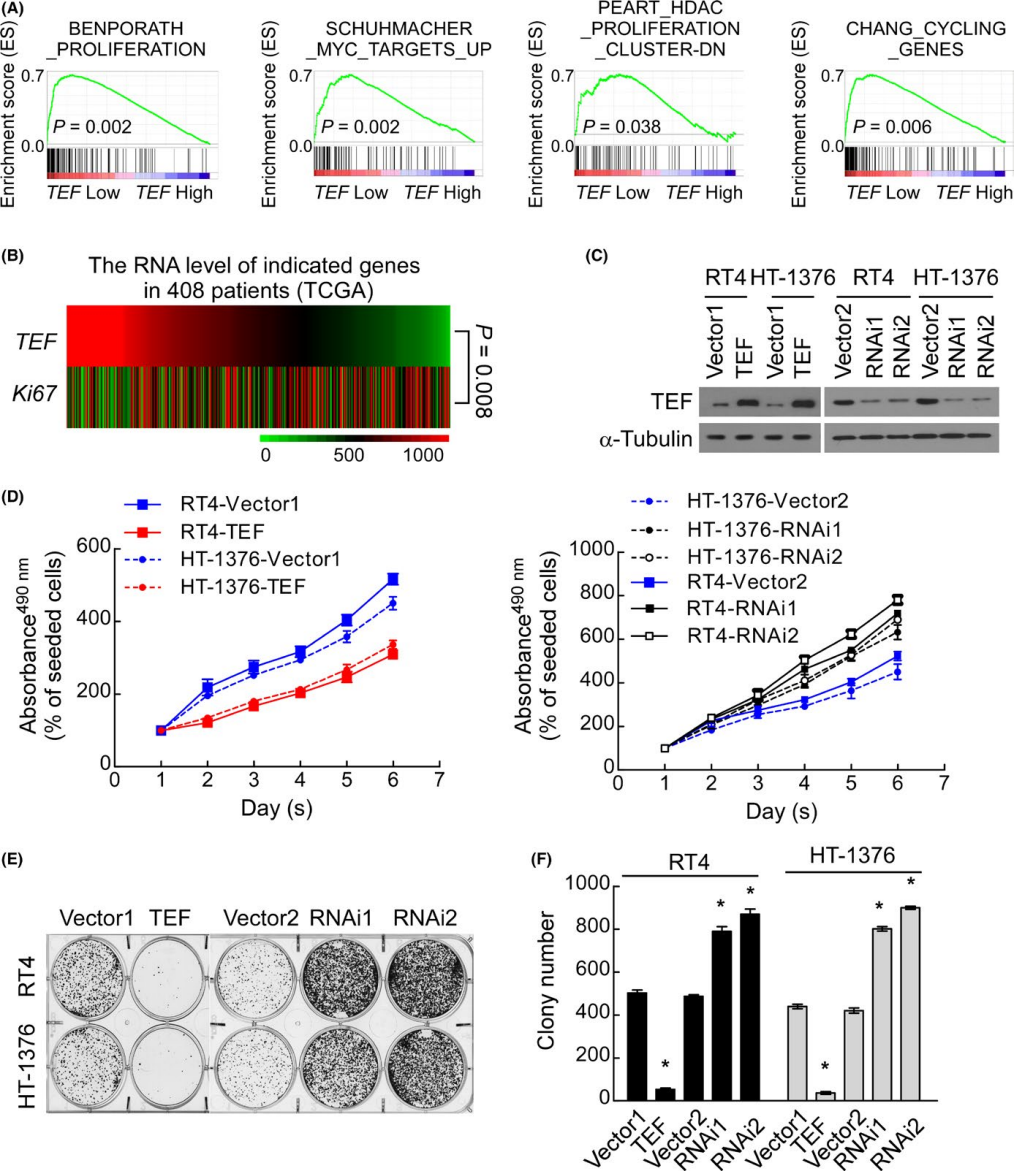
Thyrotroph embryonic factor (TEF) modulates the proliferation of BC cells. (A) GSEA plots of published data (TCGA) demonstrated a positive correlation between *TEF* mRNA expression and proliferation signatures. All *P* < 0.05. (B) mRNA expression analysis of TCGA data demonstrated a negative correlation between *TEF *and the proliferation marker *Ki67*. *P *= 0.008. (C) Western blot analysis of the indicated BC cells transfected with TEF‐vector (Vector1), TEF, TEF‐RNAi‐vector (Vector2), TEF‐RNAi1(RNAi1) or TEF‐RNAi2 (RNAi2). Three independent experiments were conducted. (D) MTT assays showed that TEF overexpression significantly decreased the growth rate of RT4 and HT‐1376 cells, while TEF downregulation significantly increased these rates. *P *< 0.05. (E) A representative image from the colony formation assay showed that TEF overexpression significantly decreased the colony number, while downregulation of endogenous TEF increased the colony number. (F) The mean count of the colony number in the colony formation assay. Data represent the mean ± SD, *P < 0.05 for TEF vs Vector1; RNAi(s) vs Vector2

Gene set enrichment analysis was used to explore the role of TEF in the above 408 BC cases and demonstrated that *TEF RNA* levels negatively impacted cell cycle regulatory genes (Figure 2A, all *P* < 0.05).^30^, ^31^ Further analysis showed that the RNA level of *TEF* negatively correlates with the mRNA level of *Ki67*, a common marker of cell proliferation (Figure 2B, *P *= 0.008). To verify these results, RT4 and HT‐1376 cells were successfully and constantly transfected with *TEF* plasmids or two *TEF*‐specific shRNA plasmids to overexpress or knockdown the expression of TEF, respectively (Figure 2C). MTT assays showed that overexpression of exogenous TEF significantly decreased the growth rate of RT4 and HT‐1376 cells, while knocking down endogenous TEF increased the growth rate of RT4 and HT‐1376 cells (Figure 2D, *P* < 0.05). Additionally, upregulation of TEF significantly decreased the mean colony number in the colony formation assay, while silencing TEF significantly increased this value compared to that in vector‐transfected cells (Figure 2E,F). These results indicate that TEF may inhibit the proliferation of BC cells.

### 3.3 TEF modulates the tumorigenesis of BC

**Figure 3:**
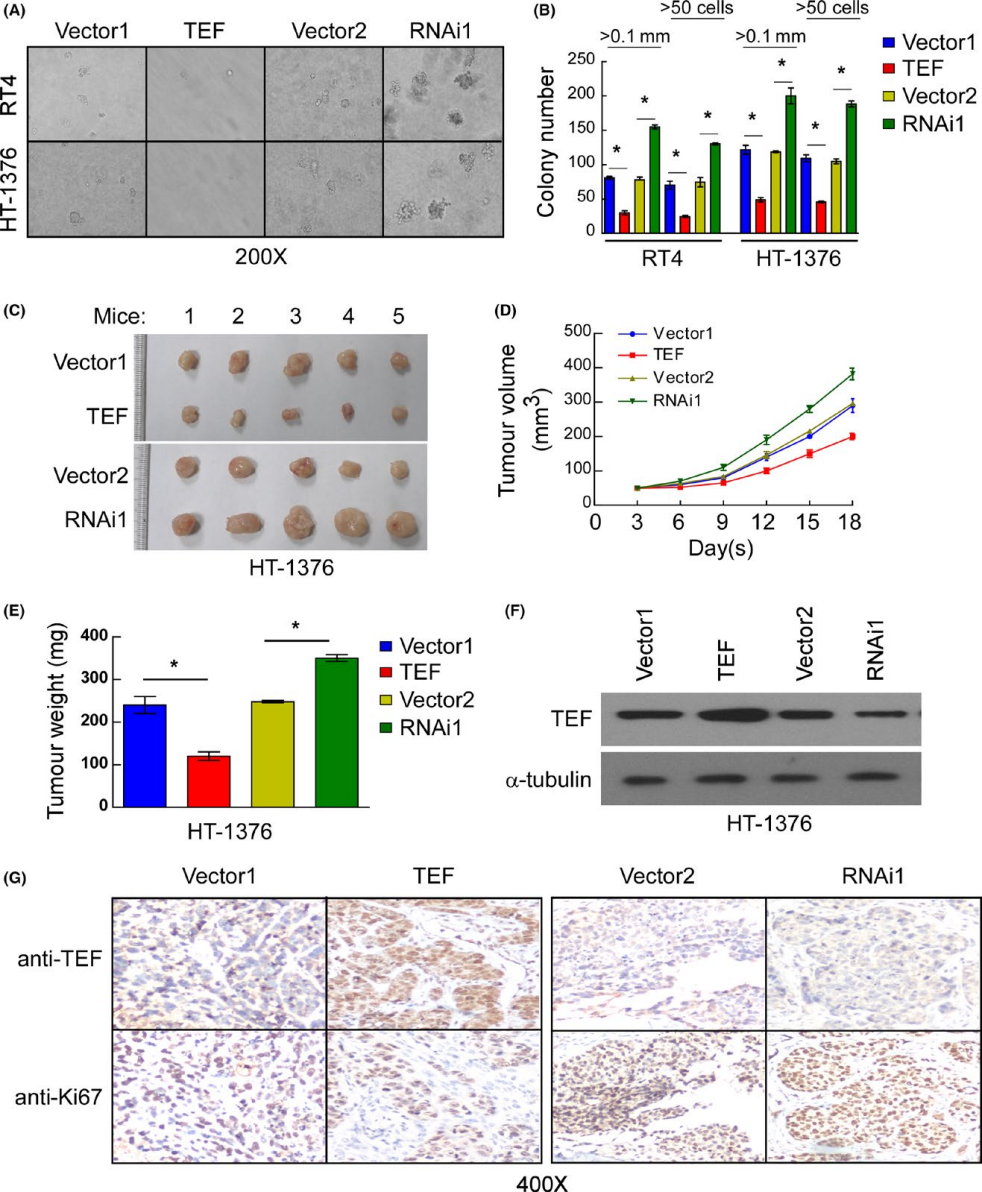
Thyrotroph embryonic factor (TEF) regulates the tumorigenesis of BC. (A and B) Representative micrographs (A) and colony numbers (B) in the anchorage‐independent growth assay. Data represent the mean ± SD of three independent experiments. *P < 0.05. (C) Images of excised tumours from five NOD/SCID mice 18 d after injection with HT‐1376‐Vector1, HT‐1376‐TEF, HT‐1376‐Vector2, or HT‐1376‐TEF‐RNAi1 cells. (D) Tumour volumes were measured every 3 d. Data represent the mean ± SD of three independent measures. (E) Average weight of excised tumours from three different weighing instruments. Data represent the mean ± SD of three independent measures. (F) Western blot analysis of TEF expression in excised tumours demonstrating that TEF expression was maintained in the tumour xenografts compared to that in the original injected cells. Three independent experiments were conducted. (G) Representative images of sections sliced from the indicated tumours and stained with anti‐TEF and anti‐Ki67. *P < 0.05 for TEF vs Vector1; RNAi1 vs Vector2

As shown in Figure 3A,B, the colony number was significantly decreased in TEF‐overexpressing cells but increased in TEF‐silenced cells, thus showing that TEF has an inhibitory effect on the malignancy of both RT4 and HT‐1376 cells when treated as a suspension (*P* < 0.05).

Additional in vivo tumorigenesis assays were performed to validate the results from the anchorage‐independent growth assay. A xenograft model in NOD/SCID nude mice created with HT‐1376 cells was used to examine the function of TEF in tumorigenesis. After comparing the tumour growth curves and final xenograft tumour weights, TEF‐overexpressing cells exhibited a more significantly decreased ability to form tumours in nude mice than vector‐transfected cells (Figure 3C‐E). Western blot analysis confirmed that TEF protein remained high in tumours generated from TEF‐overexpressing HT‐1376 cells, while TEF proteins remained low in tumours generated from TEF‐silenced HT‐1376 cells (Figure 3F). After tumour imaging, the larger part of each final xenograft tumour was paraffin‐embedded, sliced and stained. As shown in Figure 3G, the protein level of Ki67 was low in samples with high TEF protein expression and high in samples with low TEF protein expression. Thus, the TEF protein expression level negatively correlates with the protein expression of Ki67 (Figure 3G). Taken together, these results suggest that TEF plays an important role in the tumorigenicity of BC cells.

### 3.4 TEF arrests the cell cycle G1/S transition in BC cells

**Figure 4:**
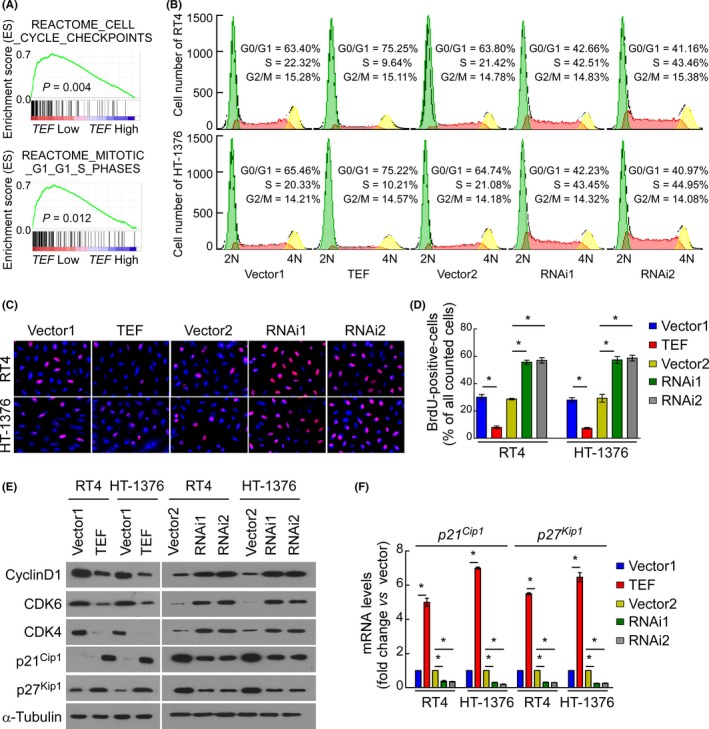
Thyrotroph embryonic factor (TEF) is involved in the cell cycle G1/S transition. (A) GSEA plots of published TCGA data demonstrated a positive correlation between *TEF* mRNA expression and cell cycle check point signatures, especially in the G1/S phase. (B) Flow cytometric analysis of stable cell lines constructed from RT4 and HT‐1376 cells demonstrated that the percentage of S phase cells negatively correlated with the expression of TEF. (C and D) Representative micrographs (C) and quantification (D) of BrdU incorporation in stable cell lines constructed from RT4 and HT‐1376 cells. Three independent experiments were conducted. (E) Western blot analysis of TEF and G1/S transition‐associated genes. (F) RT‐qPCR analysis of *p21^Cip1^* and *p27^Kip1^* expression in stable cell lines constructed from RT4 and HT‐1376 cells. *P < 0.05 for TEF vs Vector1; RNAi(s) vs Vector2. Data represent the mean ± SD

Gene set enrichment analysis also revealed that the mechanism responsible for the anti‐proliferative effects of TEF involves the G1/S checkpoint of the cell cycle (Figure 4A, all *P* < 0.05). Flow cytometry analysis showed that the overexpression of TEF significantly increased the percentage of cells in the G0/G1 phase but decreased that in the S phase. Conversely, TEF silencing significantly decreased the percentage of cells in the G0/G1 phase and increased that in the S phase (Figure 4B). As shown in the representative images of Figure 4C and the statistical diagram in Figure 4D, the percentage of BrdU‐incorporated cells dramatically decreased upon overexpressing TEF but significantly increased upon silencing TEF. These results clearly show that TEF blocks the G1/S transition of the cell cycle in BC cells.

To further investigate the biological function of TEF in arresting the cell cycle, genes closely related to the G1/S transition were examined by Western blot. As shown in Figure 4E, the protein expression levels of the cell cycle promoter cyclin D1, CDK4 and CDK6 were downregulated in TEF‐overexpressing cells and upregulated in TEF‐silenced cells. Conversely, the p21^Cip1^ and p27^Kip1^ protein expression levels were increased in TEF‐overexpressing cells but decreased in TEF‐silenced cells (Figure 4E). Moreover, RT‐qPCR analysis verified that the mRNA expression of *p21^Cip1^* and *p27^Kip1^* was increased in TEF‐overexpressing cells but decreased in TEF‐silenced cells (Figure 4F).

### 3.5 TEF regulates AKT/FOXOs signalling

**Figure 5:**
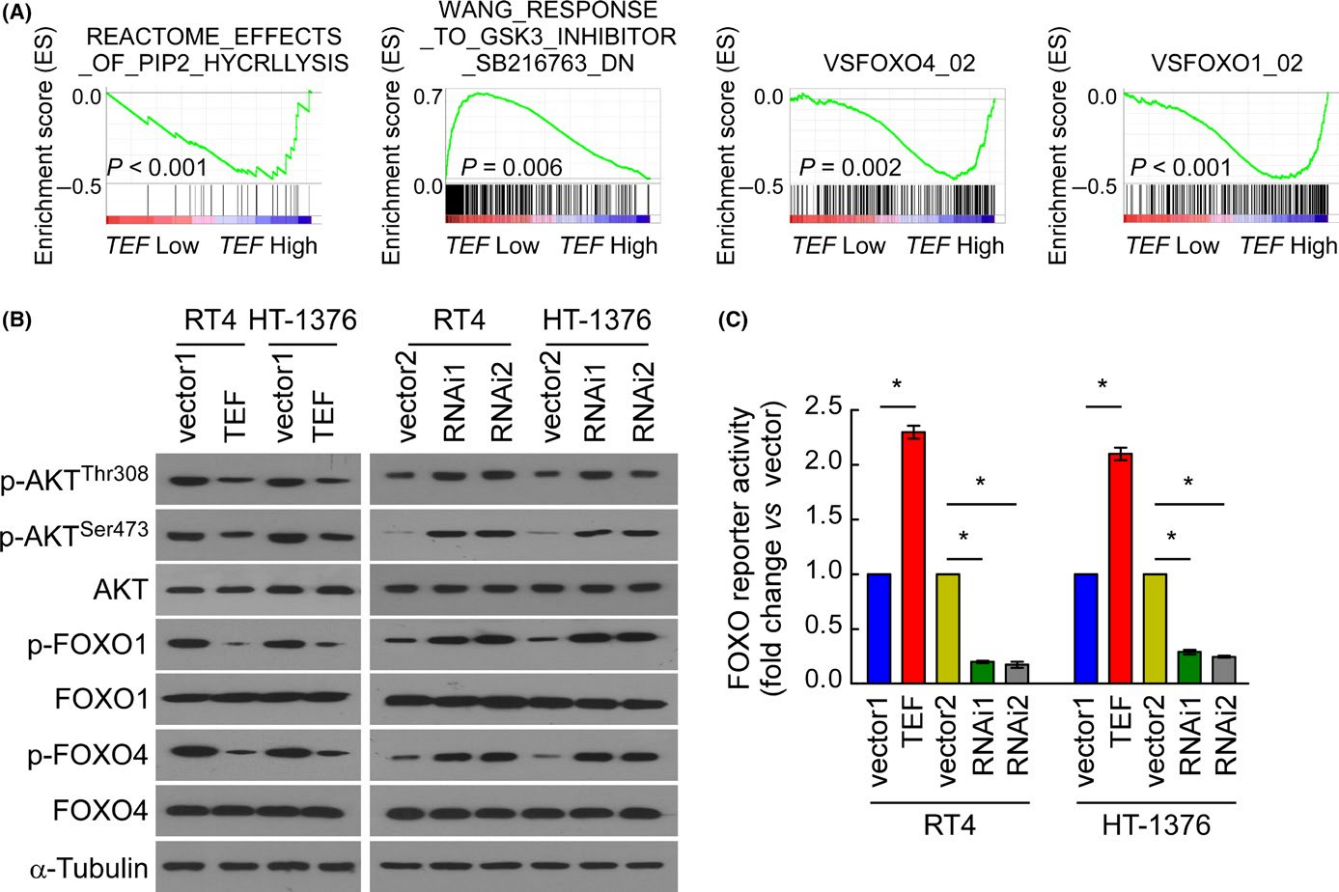
Thyrotroph embryonic factor (TEF) activates AKT/FOXOs signalling. (A) GSEA plot showing that *TEF* expression negatively correlates with AKT and FOXOs signatures. (B) Western blot analysis of AKT‐associated, FOXO1 and FOXO4 proteins in the indicated BC cell lines. (C) Relative FOXO reporter activity in the stable cell lines constructed from RT4 and HT‐1376 cells. Data represent the mean ± SD. *P < 0.05 for TEF vs Vector1; RNAi(s) vs Vector2. Three independent experiments were conducted

**Figure 6:**
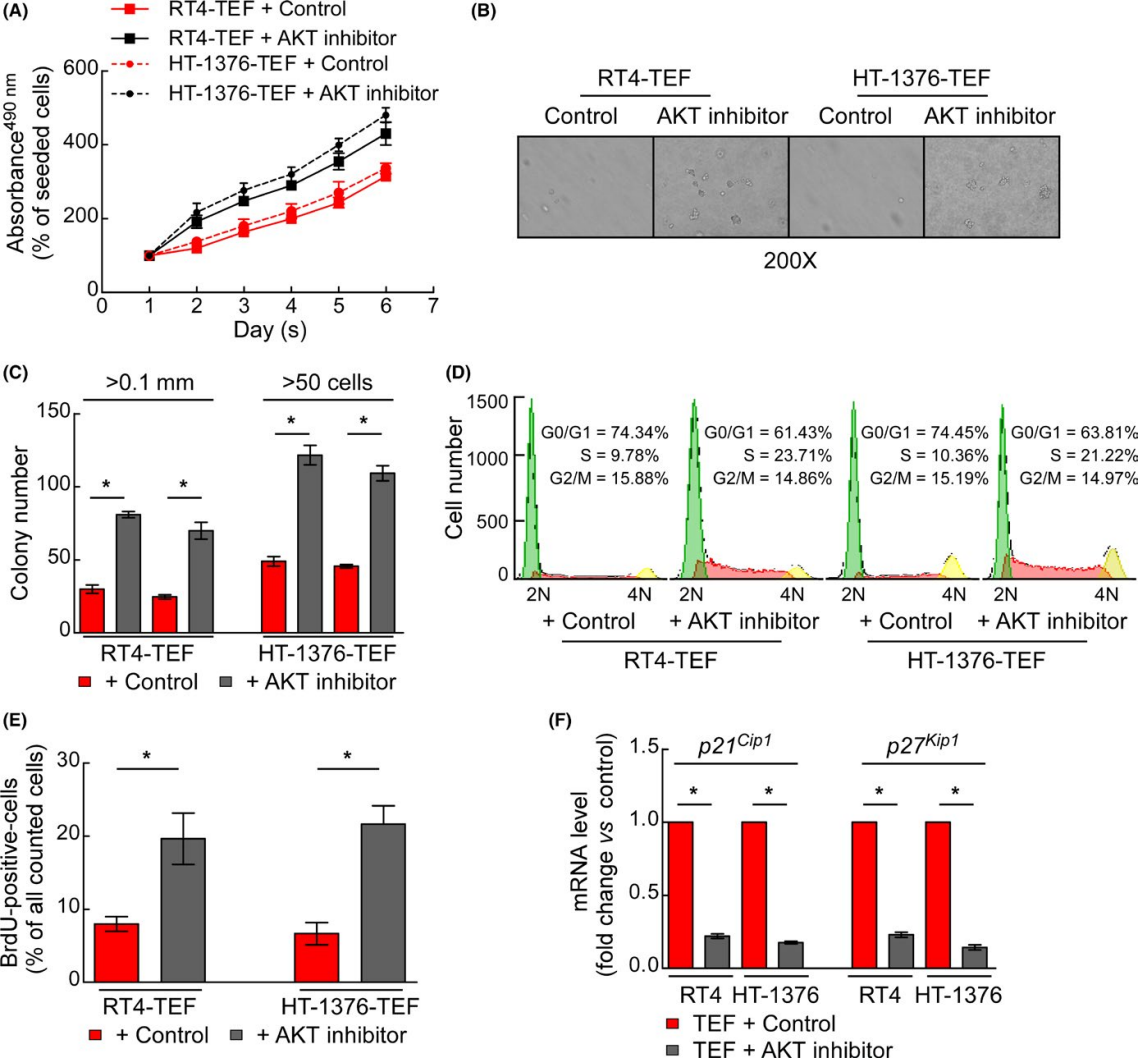
AKT inhibition attenuated the anti‐proliferative activity of thyrotroph embryonic factor (TEF) in BC cells. (A) MTT assays showing the growth rates of the constructed stable cell lines. (B) Representative micrographs of colonies in the anchorage‐independent growth assay. (C) Numbers of colonies in the anchorage‐independent growth assay. (D) Flow cytometric analysis of the constructed stable cell lines. (E) Quantification of BrdU incorporation in the constructed stable cell lines. (F) The mRNA levels of *p21^Cip1^* and *p27^Kip1^* were determined by RT‐qPCR. *P < 0.05 for the AKT inhibitor vs control. Data represent the mean ± SD. Three independent experiments were conducted

Gene set enrichment analysis was also used to explore which intracellular signalling pathway(s) might be involved in TEF‐mediated biological processes. As shown in Figure 5A, the *TE*F RNA level negatively correlated with AKT‐activated gene signatures and positively correlated with FOXO1/FOXO4‐activated gene signatures, suggesting that AKT/FOXOs signalling may be involved in the regulation of TEF. The phosphorylation of AKT, FOXO1 and FOXO4 was decreased in TEF‐overexpressing cells but increased in TEF‐silenced cells (Figure 5B). As shown in Figure 6C, the luciferase reporter assay demonstrated that the transcriptional activity of FOXOs was indeed increased in TEF‐overexpressing cells and decreased in TEF‐silenced cells. Taken together, these results suggest that the observed cell cycle arrest induced by TEF is associated with AKT kinase activity, which subsequently modulates the transactivation activities of FOXO factors.

### 3.6 AKT/FOXOs pathway is involved in the anti‐proliferative activity of TEF

To further explore the relationship between AKT and the anti‐proliferative activity of TEF, TEF‐overexpressing RT4 and HT‐1376 cells were treated with the AKT inhibitor perifosine to suppress the kinase activity of AKT. As shown in Figure 6A, the growth rate of perifosine‐treated cells overexpressing TEF was significantly increased, but the growth rate was essentially unchanged when the same cells were treated with a control. The images and histogram obtained from a soft agar assay also gave similar results (Figure 6B,C). In terms of cellular mechanisms, flow cytometry analysis showed that perifosine significantly abrogated the TEF‐mediated reduction of cells in the S phase (Figure 6D). BrdU labelling experiments also demonstrated that perifosine antagonizes the inhibitory effect of TEF protein on cell proliferation (Figure 6E). Moreover, perifosine abrogated the effects of TEF on the expression of *p21^Cip1^* and* p27^Kip1^* (Figure 6F). Our results suggest that AKT/FOXOs pathway mediates the anti‐proliferative effect of TEF in BC cells.

## 4 DISCUSSION

Many transcription factors play key roles in cellular differentiation and the delineation of cell phenotypes.^32^ Hunger et al^33^ found that TEF protein and HLF protein share indistinguishable DNA‐binding and transcriptional regulatory properties, suggesting that TEF protein and HLF protein have similar biological functions. Ectopic HLF protein expression is believed to inhibit cell death in both mouse epidermal JB6 cells and human keratinocytes.^34^ The expression of an *E2A*‐*HLF* fusion gene in acute lymphoblastic leucemia induced T‐cell apoptosis and the arrest of B‐cell maturation in vivo.^35^ All these studies indicate an important role of TEF protein in the cell growth process, especially in malignant cells. Gutierrez et al^22^ demonstrated that TEF protein expression is controlled by p53 via the upregulation of microRNA‐125b. As a tumour suppressor gene, *p53* is mutated in more than 50% of malignant tumours and regulates proliferation in various tumour cells.^36^ These findings strongly suggest that TEF protein plays an important role in the proliferation of malignant cells. In the present study, we showed that both TEF mRNA and protein expression are downregulated in BC cells and tissues. Upregulating the expression of TEF significantly retarded BC cell growth via inhibiting the G1/S transition in the cell cycle and reduced tumorigenesis both in vitro and in vivo. Meanwhile, analysis of TCGA data showed that downregulation of *TEF* RNA levels in BC patients correlates with poor survival. RT‐qPCR and Western blot analyses consistently showed that the expression of cell cycle inhibitors p21^Cip1^ and p27^Kip1^ were increased in TEF‐overexpressing cells but decreased in TEF‐silenced cells. In this study, we revealed the molecular mechanism of TEF action in malignant cells for the first time, which provides a basis for further studying its biological function.

In terms of molecular mechanisms, we found that AKT/FOXOs signalling is regulated by TEF. The mammalian forkhead transcription factors in the O class, including FOXO1 and FOXO4, are considered to be tumour suppressors due to their proapoptotic and anti‐proliferative effects.^37^ FOXO proteins translocate to the nucleus and upregulate a series of target genes, such as certain cyclin‐dependent kinase inhibitors (*p21^Cip1^* and *p27^Kip1^*). Clearly, the expression of FOXOs closely correlates with the clinicopathological characteristics and prognosis of various cancers and plays important roles in almost all types of tumours.^38^, ^39^ In diffuse large B‐cell lymphoma, FOXO4 protein is related to stem cell‐like properties and resistance to treatment.^41^ In clear‐cell renal carcinoma cells, overexpression of FOXO4 induces cellular apoptosis.^42^ In BC, FOXO1 protein was reported to mediate cell apoptosis.^43^ Other biological events, such as autophagic flux, oxidative stress, self‐renewal, migration and invasion, were also demonstrated to be regulated by FOXOs.^44^, ^45^ However, mechanistic studies on FOXOs are rare and tend to focus on several key biological molecules, among which the most well‐characterized and important is the Ser and Thr kinase AKT (also known as protein kinase B, PKB).^48^ One direct piece of mechanistic evidence is that FOXOs protein phosphorylation is enhanced in the growth plates of AKT transgenic mice.^49^ FOXO transcriptional effectiveness is largely determined by phosphorylation‐dependent nucleocytoplasmic shuttling.^50^ AKT phosphorylates FOXOs, which leads to their translocation to the cytoplasm, thereby promoting the proliferation of malignant cells.^51^ This research shows that the phosphorylation of AKT, FOXO4 and FOXO1 is decreased in TEF‐overexpressing cells but increased in TEF‐silenced cells. Furthermore, a luciferase reporter assay demonstrated that the transcriptional activity of FOXO was increased in TEF‐overexpressing cells and decreased in TEF‐silenced cells. These results suggest that TEF protein regulates AKT/FOXOs signalling; however, the mechanism underlying these effects remains unclear. In glioma, HLF protein is directly bound to the miR‐132 promoter to enhance the expression of miR‐132. PTEN, a powerful regulator of AKT, is a target of miR‐132. The binding partners of TEF are similar to those of HLF, indicating that TEF protein might regulate the activity of AKT by controlling the expression of micro‐RNAs. This hypothesis is a topic worthy of further research.

Taken together, this study shows that TEF is downregulated in BC cells and plays an important role in suppressing proliferation and tumorigenesis by deactivating AKT/FOXOs signalling and suggests a potential role of TEF as a diagnostic marker and valuable therapeutic target in BC.

## CONFLICT OF INTEREST

The authors have no conflicts of interest to declare.
